# UNIPDES - An internet-based transdiagnostic intervention for college students’ psychological symptoms: Evaluation of its development, usability and effectiveness: Study protocol

**DOI:** 10.1016/j.conctc.2025.101443

**Published:** 2025-02-06

**Authors:** Ömer Özer, Gizem Öztemür, Ali Ercan Altinöz, Burak Köksal, Uğur Doğan, Sedat Batmaz, Recep Gür, Ahmet Altinok

**Affiliations:** a Department of Guidance and Psychological Counseling, Anadolu University, Eskisehir, Turkey; b Department of Guidance and Psychological Counseling, Middle East Technical University, Ankara, Turkey; c Department of Psychiatry, Eskisehir Osmangazi University, Eskisehir, Turkey; d Department of Guidance and Psychological Counseling, Tokat Gaziosmanpasa University, Tokat, Turkey; e Department of Guidance and Psychological Counseling, Mugla Sitki Kocman University, Mugla, Turkey; f Department of Psychology, Ankara Social Sciences University, Ankara, Turkey; g Department of Educational Sciences, Eskisehir Osmangazi University, Eskisehir, Turkey; h Department of Psychology, University of Groningen, Groningen, Netherlands

**Keywords:** Internet-based intervention, Transdiagnostic cognitive behavioral therapy, University students

## Abstract

University students often face significant mental health challenges, including depression, anxiety, and difficulties in adjustment, which can be exaggerated by the demands of independent living and increased life responsibilities. These challenges are often compounded by barriers to seeking help, such as stigma and limited access to university resources, which can further deteriorate students' well-being. This protocol was created to assist college students in overcoming these obstacles and to assess, in comparison to a control group, the impact of a guided and unguided online intervention platform based on transdiagnostic CBT (UNIPDES) on depression, anxiety, and adjustment levels. The calculated sample size for the study will include 330 students, and the participants will be selected from five different universities located in Türkiye. Participants will be randomly assigned to either guided, unguided, or control groups. Guided and unguided group participants will receive six weeks of intervention, and the waitlist control group will receive the unguided version of the program after twelve weeks of randomization. Assessments will take place at baseline, post-test (8 weeks post-baseline) and follow-up (12 weeks post-baseline). A Mixed ANOVA will be employed to analyze the data, with Group (Guided, Unguided, Control) as the between-subjects factor and Time (Baseline, Post-Test, Follow-Up) as the within-subjects factor, as well as to assess the interaction effect between Group and Time on the primary outcomes—changes in depression, anxiety, and adjustment levels. Additionally, students’ reasons for dropout will be assessed qualitatively. The results from this study can build evidence for the effectiveness of transdiagnostic guided and unguided internet-based intervention for treating depression, anxiety, and adjustment problems of students. UNIPDES can provide a flexible, easy-to-access, and cost-effective treatment for the problems that students commonly face. Trial registration is registered at ClinicalTrials.gov Protocol Registration and Results System (Trial number: NCT06245200).

## 1 Introduction

The university years represent a shift from youth to adulthood and are characterized by increased independence in handling life's challenges and making decisions which leads to heightened emotions of responsibility and the need to make decisions about potential romantic partners and career possibilities [1]. Owing to developmental alterations in several spheres of life, these years are crucial for people's mental and overall well-being [2] and also critical for marking the onset of several mental health problems [3,4]. Previous studies [3,[5], [6]] have shown that university students deal with a range of challenges in their personal, professional, academic, and social lives and these are found to be closely related to several mental health issues, especially stress, anxiety, and depression. According to a survey, students who were having trouble adjusting to school, managing their workload, their mental health, and their requirements for development were the main reasons they were reaching out to the university's counseling services [7].

A growing number of university students are experiencing psychological problems [[8], [9], [10]] and these have been increasing exponentially over the years [11,12]. These psychological problems especially affect students in the academic field [13,14] and are closely related to higher dropout rates [5].

A number of contextual and personal factors were involved when discussing university students' mental health. Byrd and McKinney [15] found that improved coping skills, communication skills, a strong spiritual identity, academic self-confidence, heterosexuality, intergroup awareness, social bonds, and satisfaction with the school have a positive impact on college students' mental health. Suicidal thoughts, difficulty balancing work and school, a negative disposition toward the university environment, and less engagement with instructors were, nonetheless, linked to poor well-being. Similarly, in their systematic review, Campbell et al. [16] found that several factors such as sexual orientation, ethnicity, gender, previous mental health problems, childhood traumas, previous experiences with parents (i.e., overcontrolling parenting), and having a disability were related to poor mental health, whereas optimism, mental health literacy, having strong social support, participation in learning, good experiences and involvement in lectures, and self-efficacy were associated with better mental health.

Although university students often experience psychological problems, they are reluctant or unable to seek professional help for various reasons, such as a sense of self-sufficiency, fear of stigma, or privacy concerns [17]. On the other hand, insufficient resources of university counseling centers cause long waiting lists for students seeking help [18]. When these elements are evaluated together, there is a need for alternative options that are permanent, appeal to students, can reach large audiences, and can eliminate the obstacles that university students face in seeking psychological help.

### 1.1 Internet-based interventions for mental health

Internet-based interventions can be either unguided (self-directed, without input from a therapist) or guided (with varying degrees of therapist contact). These programs are designed to raise awareness of psychological issues and provide guidance, treatment, and support to individuals suffering from various psychological problems ([19,20] Digital Health, 2022). Recent experimental studies have shown that internet-based interventions are useful for a variety of problems, including depressive mood [21,22], stress management [23], social anxiety [24,25], obsessive-compulsive disorder [26,27], substance and alcohol use [28,29], and suicide prevention [30]. Previous studies on the effectiveness of internet-based interventions with university students also showed promising results in reducing problematic internet use [31], alcohol and drug addiction [32,33], and depressive mood and anxiety [34]. The abovementioned studies either have guided and unguided groups and control group, or one group (i.e., guided) and control group when comparing the effectiveness of internet-based interventions, and the effectiveness of the internet-based interventions were evaluated over participants’ improvements on measured problem areas. The results, overall, suggested that internet-based interventions can be as effective as traditional therapies in decreasing symptoms on problem areas. When the literature has been examined regarding the differences in effectiveness of guided and unguided methods of delivery of internet-based interventions, an umbrella review of meta-analyses by Zhang and colleagues [35] reported that the results of guided interventions typically demonstrate more effectiveness than unguided ones.

Internet-based treatments offer several benefits, such as being accessible at any time or place, lowering stigma by preserving anonymity, lowering the need for trained specialists and associated costs by reaching many people at once, and letting individuals move at their own pace. It is clear that internet-based interventions are preventive in the sense that they provide information about mental health, encourage help-seeking, reach a large number of people at once, reduce the symptoms of the individual's mental health problem, increase their well-being, and prevent mental disorders from persisting in the future [36]. Besides their advantages, dropout rates denote a significant challenge which reduces their effectiveness and sustainability (e.g., Ref. [37,38]). Therefore, understanding the attitudes towards internet-based interventions is crucial due to their effects on dropout rates. Specifically, understanding how positive or negative attitudes influence individuals' decisions to adhere to or discontinue such interventions can provide valuable insights for improving the design and implementation of these approaches.

One of the main sources that students get psychological help is the guidance and counseling centers (GRC) of the universities (i.e., [39]). In Türkiye, the guidance and counseling services in universities has begun for the first time in 1973 by a higher education law number 1750. In 1982, by another law, universities have been obligated to establish GRCs [40]. University GRCs includes professionals from psychiatry, counseling, psychology and social work and these centers provides variety of services (i.e., individual counseling, group counseling, consultancy), and one of the biggest concerns of staff working on these GRCs is the work overload [41]. On the other hand, internet-based interventions are a relatively new field of study in Türkiye. Previous research on this area consisted of compilations and reviews [42,43]. Several internet-based intervention programs included an unguided self-help program for psychological symptoms during COVID-19 [44,45], cognitive distortions and psychological symptoms for children [46], dealing with COVID-19 distress [47], obsessive-compulsive disorder [48,49]. In all these interventions, a rather limited/moderate sample size was used.

The transdiagnostic approach (TA) focuses on the common psychopathological processes that underpin the development and persistence of mental disorders. Even if various problems are classified differently, it is possible to discern how they overlap or differ from one another, and TA tries to treat mental disorders based on these overlapping elements [50]. TA has a flexible and modular structure that allows it to be easily integrated into the cognitive behavioral therapy (CBT) process [51,52]. Transdiagnostic treatments in CBT include universal strategies that can be used across problems. Several strategies, thus, utilized within TA includes targeting explicit selective memory, emotional inference, repetitive negative thinking style, emotional attention to external and internal stimuli, attention focused on avoidance and security, positive and negative metacognitive beliefs, avoidance behaviors, safety seeking behaviors, and experiential avoidance [53,54]. TA, therefore, is a framework for doing therapy in a transdiagnostic and effective manner, while others are disorder-specific. Its clarity, simplicity, and focus on commonality make it effective in treating comorbidities and addressing various problem areas [55].

Previous studies showed the effectiveness of internet-based TA intervention on people with anxiety and depression [[56], [57], [58]], sleep-related problems for adults and adolescents [59], and studies with college samples found that TA-based internet-based interventions were effective in treating underlying personality risk factors [60], depression [34], anxiety and depression [61]. All these programs were presented with a guide (rather than self-help only). Even if follow-up studies showed that the benefits of interventions persist [61], the literature also states the requirement for further research to identify the mechanism behind their effectiveness [62].

### 1.2 Aims

This study aims to accomplish two main objectives. The initial goal is to test the effectiveness of the internet-based intervention for university students, UNIPDES, which is based on a transdiagnostic CBT, in raising psychological adaptation levels and improving the depressive and anxiety symptoms in university students, controlling for participants' initial depressive symptoms, anxiety, psychological adjustment scores, and attitudes toward internet-based interventions. Consistent with this objective, we seek to evaluate the variations between guided and unguided treatment delivery compared to the waiting list control group.

The second goal is to assess the reasons why users who enroll in internet-based interventions dropout. In order to assess the possible influence of participant attitudes on internet-based therapies on dropout rates, we want to incorporate an analysis of these attitudes into our study. In particular, knowing how people's attitudes, whether favorable or unfavorable, affect their choices to stick with or stop using such interventions can help a researcher to improve their design and execution. Our study aims to add to the body of knowledge on dropout in internet-based interventions in this regard.

## 2 Methods

### 2.1 Study design

**Fig. 1:**
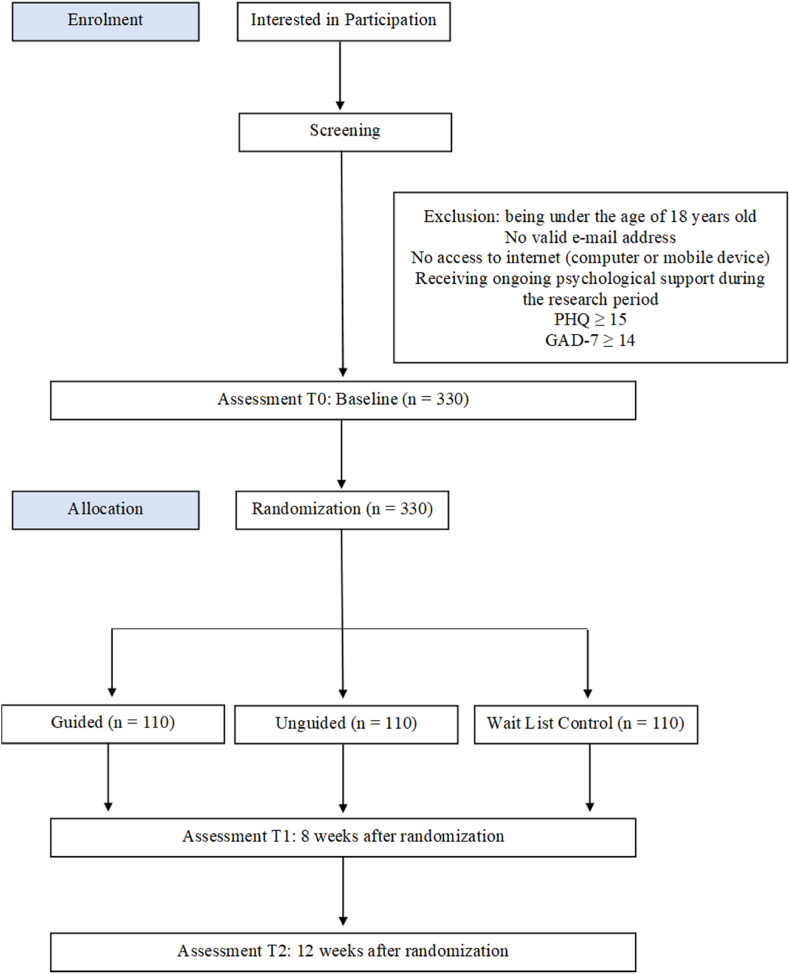
Consort flow-chart of the study.

This project was designed as a mixed methods study to investigate the impact of an online intervention program based on the transdiagnostic CBT on university students' levels of psychological adaptation, reduction of depressive mood, and anxiety symptoms, as well as the durability of these effects [63]. The rationale behind utilizing a mixed method is typically that combining qualitative and quantitative data yields a more comprehensive grasp of the study problem than each approach could on its own [64]. Developing an online intervention platform based on transdiagnostic CBT (UNIPDES) and evaluating its effectiveness are the primary objectives of the research. The clinical Research Ethics Committee of the third author's university approved the study protocol (approval number E−80558721-050.99-2300154653), information booklet, and informed consent form. The trial is registered on clinicaltrials.gov (Trial Number:NCT06245200) and the CONSORT chart (Fig. 1) was presented on how the study is conducted and reported.

### 2.2 Participants

Participants in this study will be composed of students from five different universities located in Türkiye. All these universities are public universities, and they range from small (around 5000 students) to large scale (around 1.000.000 students) based on their student numbers. Participants who took part in the quantitative study will also be the ones chosen for participation in the qualitative part. The following inclusion criteria will be used for participation: a) volunteering to participate in the study; b) having a valid e-mail address; c) having internet access (computer or mobile device); d) being over the age of 18; e) not receiving any ongoing psychological support during the research period; and g) scoring less than 15 on the Patient Health Questionnaire 9 (PHQ-9) and less than 14 on the Generalized Anxiety Disorder-7 (GAD-7) scales.

In line with our aim of developing a preventive program (UNIPDES) designed for a broader audience, the majority of participants in our study are university students and do not represent a clinical sample. Only individuals with severe problems were excluded from the study, as these individuals are more appropriate for individualized psychological counseling for their needs than internet-based interventions. Therefore, this study aims to reach a heterogeneous sample, which results in addressing the needs of a wider population and creating a program with preventive and protective features.

### 2.3 Procedure

Posters and online announcements of the study will be created and sent to the five universities mentioned above. The project website, advertising posters, academic institutions' websites, and social media sites will all be used to announce the study. Students will be able to navigate to the project website by scanning the QR code on the posters.

Upon accessing the project website, participants will be asked to complete an online informed consent form outlining the study's aims before deciding whether to volunteer. After obtaining the participants' consent to volunteer in the study, they will be requested to fill out the questionnaires. Those who meet the remaining inclusion requirements but score highly on PHQ-9 and GAD-7 or undergoing psychological treatment will not be able to register or access the system. These users will be notified to seek professional help if they so choose, along with a window with contact details for resources (counseling center of the university, medico-social units) that will enable them to receive help at the appropriate university. Allotted participants will get e-mails and SMSs letting them know which group they are in. To guarantee the validity and reliability of our results, we want to take this heterogeneity into consideration during our analysis procedures. The consort flow chart of the study is presented in Fig. 1.

In the qualitative part, to ensure diversity in the semi-structured interview process, participants who discontinued the program with the lowest and highest symptom levels will be invited, while maintaining a balanced gender ratio. This stratified approach aims to capture a wide range of perspectives. The interviews will be conducted by researchers holding doctoral degrees in psychology, ensuring both expertise and consistency in data collection.

### 2.4 Randomization

Through the use of software (randomizer.org), participants who have registered on the project website and fulfilled the inclusion criteria will be assigned to groups randomly. Care will be given to guarantee that the distribution of men and women is equal to achieve maximum variety in each group. Therefore, the randomizer.org website's block approach will be utilized. Participants will remain blinded to their group assignment to minimize bias. Group assignment will be managed by a third party using coded identifiers. Participants in the intervention group will immediately access the program, while those in the waitlist control group will be informed that the program will not start immediately for them but will be made available after a waiting period. Both groups will receive equivalent instructions and communication to ensure they cannot discern their allocation. This approach balances expectations and perceived fairness, reducing potential bias in participant responses.

### 2.5 Sample size estimation

The G∗Power program [65] was utilized to determine the sample size. The program's objectives included three groups, three assessments, four covariates (depression, anxiety, adjustment, and attitude toward internet-based interventions score), a medium effect size (Cohen's *d* = 4, Cohen's *f* = 2), a power of 80 %, and a significance level of *p* = 0.05. Therefore, a minimum sample size of 244 individuals is needed to detect a significant difference.

It is important to consider dropout rates while determining the sample size. Based on their review from the studies between 1990 and 2009, Melville et al. [37] found that the dropout rates range from 2 to 83 %, with an average rate of 31 % in internet-based interventions. Another meta-analysis by Meyerowitz-Katz and colleagues [38] reported the average attrition rate in internet-based interventions as 43 % in studies published in between 2011 and 2019. Based on the previous research (also see Ref. [66,67]), a 35 % dropout rate is anticipated for this study. The dropout rates for internet-based interventions are comparable to those in previous studies based on traditional counseling interventions [68], in their meta-analysis, reported that 55 % of total participants completed the internet-based treatments.

In light of the power analysis calculated through G∗power software, it is suggested that each group should contain 81 people. Due to our expectation of a 35 % dropout rate, an additional 30 participants will be added to each group.

### 2.6 Intervention development

The intervention program builds on a transdiagnostic CBT approach. The TA was preferred because it has a flexible and modular structure that can be easily integrated into the functioning of CBT [51,52]. The intervention program is founded on this approach and the relevant literature [34,51,58,60,69,70]. The intervention will consist of six modules. The first module's topics will be goal setting and motivation enhancement. Psychoeducation and mindfulness will be covered in the second module. The third module will focus on cognitive restructuring and other cognitive interventions. Exposure and other behavioral interventions will be the main topics of the fourth module. The fifth module will include emotion regulation strategies. Finally, the last and sixth modules will be devoted to relapse prevention strategies and termination.

### 2.7 Guidance

After random assignment of participants into three groups (guided, unguided, or control), participants in the guided group will receive expert-written feedback within 24 h of each module's completion. Feedback will be delivered by researchers (guides) working on the project as scholars and these researchers/guides will receive two days of training from two specialists who are experts in CBT and TA. The researchers (guides) working on this project have been educated in psychology/counseling fields (at least a master's degree) and have previous experience in the CBT approach. All the feedback provided by guides to program users will not exceed 300 words. The feedback will not include therapeutic intervention beyond what is offered in the program, as the feedback's main goal is to encourage/motivate the participant. The other/next module will start after the feedback message has been sent. Before sending feedback to participants in the guided group, all the feedback composed by researchers/guides will be checked and approved by those who train guides to maintain consistency across researchers/guides.

Participants in the unguided group will utilize the program entirely on their own and will not receive regular feedback upon module completion. The next module will be available the day after the previous one is finished. Participants in both guided and unguided groups will receive reminder messages on the third and fifth days if they have not yet begun the next module in the program.

### 2.8 Control condition

The waitlist control (WLC) group will be asked to complete pre- and post-assessments at initial registration and by the end of the eighth week. Participants will not be in contact with the research team throughout the waiting period. They will be given the unguided version of the program at the 12th week, after the completion of the assessment tools. Participants who complete all measurement tools will receive a gift card (which they can spend on numerous web pages) as a means of encouragement.

### 2.9 Measurements and outcomes

Three assessments will be conducted as part of the study: baseline (T0) for all groups, immediately following program completion (T1; 8 weeks), and a follow-up four weeks following randomization (T2; 12 weeks). All participants, regardless of whether they have completed the program, will receive the assessment at T1 (8 weeks). Table 1 provides a summary of all the variables and measurements.

**Table 1:** Summary of measurement tools.

Measurement Tool	Pilot Study	T0	T1	T2
Demographic Information Form	X	X		
Patient Health Questionnaire – 9 (PHQ-9)		X	X	X
Generalized Anxiety Disorder – 7 (GAD-7)		X	X	X
The Brief Adjustment Scale – 6 (BASE-6)		X	X	X
System Usability Scale (SUS-10)	X	X		
Attitudes Toward Internet-Based Interventions Scale (ETAM)		X		
Module Evaluation (guided and unguided group)			X	
User Experience on Program Effectiveness			X	
User Experience on Dropout			X	

#### 2.9.1 Outcome measures

##### 2.9.1.1 Patient Health Questionnaire-9 (PHQ-9) Turkish form

PHQ-9 was developed by Kroenke et al. [71] to gauge the symptoms and severity of major depressive disorder. The Turkish adaptation of the scale was conducted by Sarı et al. [72]. The higher scores obtained from the scale indicate higher symptomatology. The psychometric properties of the scale showed acceptable results. The Cronbach's alpha for the Turkish version was reported as .84.

##### 2.9.1.2 Generalized Anxiety Disorder-7 (GAD-7) Turkish form

GAD-7 was developed by Spitzer et al. [73] to measure anxiety disorder symptoms. The Turkish adaptation of the scale was conducted by Konkan et al. [74]. The higher scores obtained from the scale indicate higher symptomatology. The Cronbach's alpha for the Turkish version was reported as .85.

##### 2.9.1.3 The brief adjustment Scale-6 (BASE-6) Turkish form

BASE-6 was developed by Cruz et al. [75] to gauge general psychological distress and adjustment. A Turkish adaptation of the scale was conducted by Yıldırım and Solmaz [76] with a sample consisting of university students. The internal consistency of the scale was reported as .88.

##### 2.9.1.4 Attitudes toward internet-based interventions scale (ETAM) Turkish form

The ETAM was developed by Apolinário-Hagen et al. [77] to gauge people's opinions about internet-based interventions as a substitute for traditional in-person psychotherapy. Higher scores obtained from the scale indicate more positive attitudes. The Cronbach's alpha of the scale in the original study was reported as .92. A Turkish adaptation study of the scale was conducted by Özer et al. [78] and the internal consistency was reported as .86.

#### 2.9.2 Other measures

##### 2.9.2.1 Demographic information form

A demographic form created by the researchers to gather information about the demographics of the participants, including their age, gender, current psychiatric diagnosis (if any), and history of psychiatric treatment/help.

##### 2.9.2.2 System usability scale (SUS-10) Turkish form

SUS-10 aims to assess the impact of program usability on treatment outcomes [79]. The higher scores obtained from the scale indicate better program usability. The Turkish adaptation of the scale was conducted by Kadirhan et al. [80]. Cronbach's alpha was reported as .78.

##### 2.9.2.3 Module evaluation

At the end of each module completed, the participants will be asked to rate the effectiveness of the module on a single question (0- not effective, 10-very effective). The overall program evaluation will be made based on the sum of these scores.

##### 2.9.2.4 User experience on program effectiveness

Qualitative approaches will be used to assess program participants' experiences. Following the use of quantitative measures to evaluate the participants' progress, those who demonstrated the highest and lowest levels of progress will be invited to an online, semi-structured interview. This will be determined by calculating the difference between the participants' pre-and post-test results. To find out more about the user experience, a total of 16 participants—eight from the group with the highest improvement and eight from the group with the lowest improvement—will be asked to provide information about their experiences to learn more about the effectiveness of the internet-based self-help platform. The example questions include “How was your experience with the self-help platform?" and “What were the advantages and disadvantages of the self-help platform?”

##### 2.9.2.5 User experience on dropout

Participants who completed at most two modules -or less-among those who have quit using the platform will be the subject of a semi-structured qualitative interview to learn more about the experiences that caused them to drop out early. 16 dropout participants -eight participants from the guided group and eight participants from the unguided group-will be chosen at random based on their ID numbers using the randomizer.org website. The semi-structured interview questions will focus on challenges faced while using the program, reasons for early discontinuation, and features participants believe could have encouraged them to continue. The example questions include “Can you explain the reasons why you quit using the platform?" and “What were the problems that you faced while using the platform?” Based on these key questions, the participants will be interviewed using probes to get more useful responses. This approach seeks to gather comprehensive insights to improve the program's design and accessibility.

### 2.10 Statistical analyses

A Mixed ANOVA will be employed to analyze the data, with Group (Guided, Unguided, Control) as the between-subjects factor and Time (Baseline, Post-Test, Follow-Up) as the within-subjects factor. This approach will assess both the main effects of Group and Time, as well as the interaction effect between Group and Time on the primary outcomes—changes in depression, anxiety, and adjustment levels. Post-hoc tests will be conducted to explore significant interactions. If necessary, covariates will be included in the analysis through a Mixed ANCOVA to adjust for baseline differences.

Dropout and missing data are anticipated in the study. To address this, missing data will be handled using relevant methods, e.g., multiple imputation or Multilevel Mixed-Model Regression Analysis, depending on the nature and pattern of missingness. Multiple imputation will be employed to estimate missing values if the data are missing at random, while Multilevel Mixed-Model Regression Analysis will be applied to account for both missing data and repeated measures over time. Baseline characteristics across the three groups will be tested using statistical methods such as t-tests, chi-square tests, or ANOVA to ensure comparability. Any significant differences will be adjusted for in subsequent analyses. These approaches will help ensure the robustness of the findings despite anticipated dropout and missing data.

The qualitative data will be transcribed and coded into themes by two independent researchers to ensure reliability. In the analysis of the data, the MAXQDA program will be utilized. According to the principles of [81] to ensure the reliability and validity of the results, the coding and assignment of themes will be conducted by several researchers, and the evaluation will be made through consensus/(disagreement + agreement) ∗100 formula as suggested. At the end of the analysis, consensus and disagreements will be evaluated in a project team meeting.

## 3 Discussion

The purpose of this study protocol is to outline the randomized controlled study design, which assesses the effectiveness of both guided and unguided online intervention platform based on transdiagnostic CBT (UNIPDES) on the levels of psychological adaptability, anxiety, and depressive mood in university students. It is anticipated that the participants displaying anxiety, depressive symptomatology, and challenges with psychological adaptation will benefit clinically significantly from the UNIPDES (either guided or unguided) in comparison to the waitlist control group. While the primary focus of the UNIPDES program is to provide clinical benefits for participants displaying anxiety, depressive symptomatology, and challenges with psychological adaptation, we also anticipate that participants without symptoms will experience positive outcomes. For these individuals, the program is expected to serve a preventive function, enhancing psychological resilience and promoting adaptive coping strategies for future stressors. By targeting a heterogeneous sample, we aim to evaluate the program's effectiveness not only in reducing existing symptoms but also in fostering psychological well-being and prevention among symptom-free participants. These distinctions will be considered in our analysis to assess the program's impact across different participant profiles. Furthermore, the investigation of the therapeutic implications of the effectiveness of two distinct intervention levels of guidance -guided and unguided - will enhance the value of the study.

This study has several foreseen limitations. First, the study sample will include students from selected universities located in Türkiye and is thus not representative of the general population. Another expected limitation is the sample size. Considering the possibility of a 35 % dropout rate, a larger sample size was targeted to meet the requirements needed to evaluate the effectiveness of the program. Even if the required sample size is possible to obtain, in cases of lower rates of participation, the program will be announced to students studying at different Turkish universities as an alternative. A similar plan will be followed in case the dropout rates increase more than expected (35 %). The study's use of a waiting control group constitutes another drawback. It may be more difficult to verify that the treatment alone will be the cause of the outcome because of some external factors (i.e., time, changes in life conditions) that may obscure the treatment's effectiveness. Similarly, using a control group may lead to overestimating the treatment effects, particularly due to minor improvements observed in the symptoms of participants in the control group condition [82].

Additionally, the study has several strengths. First off, most past studies (both globally and in Turkey) on internet-based interventions tended to concentrate on the treatment of particular problem areas [[45], [47], [48], [49]]. The intervention program will make use of the transdiagnostic approach (TA), which gives professionals the flexibility to target multiple problem areas instead of a single one. Even though the TA has received a lot of attention, no prior research has been done to evaluate its effectiveness in a self-help program. Thus, the use of a TA, which enables interventions to reach a wider range of problem areas, is another strength of this study. Lastly, the acceptability and usability of these interventions were neglected in earlier research assessing the effectiveness of internet-based self-help programs. Though it is well known that the main problem with internet-based therapies is premature termination (e.g., Ref. [68]), early dropout has been a significant worry that is often disregarded. As a result, this study's ability to pinpoint the causes of early user dropout will help guide future research on ways to improve the acceptability and usability of internet-based interventions.

All in all, this study will contribute to clinical practice for several reasons. As previously said, university students frequently struggle with psychological adaptation, anxiety, and depression, but they may choose not to seek help for a variety of reasons (i.e., fear of being stigmatized). On the other hand, even though university psychological counseling centers are a crucial place for students to get psychological support, students find it challenging to access these resources due to long waiting lists and the high workload of the mental health professionals. It is believed that this program will lessen the workload of professionals in this field and decrease long waiting lists while also providing students with a readily accessible, anonymous, cost-effective and self-paced alternative. Future research focusing on various age groups, different psychotherapy schools (i.e., other than TA-based CBT), and psychological problems is also anticipated to benefit from the results of this study.

## Funding

This work was supported by the Scientific and Technological Research Institution of Turkey under grant number 123K895.

## CRediT authorship contribution statement

**Ömer Özer:** Writing – review & editing, Writing – original draft, Supervision, Resources, Project administration, Methodology, Investigation, Funding acquisition, Conceptualization. **Gizem Öztemür:** Writing – review & editing, Writing – original draft, Conceptualization. **Ali Ercan Altinöz:** Writing – review & editing, Writing – original draft, Supervision, Methodology, Conceptualization. **Burak Köksal:** Writing – review & editing, Methodology, Conceptualization. **Uğur Doğan:** Writing – review & editing, Supervision, Conceptualization. **Sedat Batmaz:** Writing – review & editing, Supervision, Conceptualization. **Recep Gür:** Writing – review & editing, Supervision, Conceptualization. **Ahmet Altinok:** Writing – review & editing, Supervision, Conceptualization.

## Declaration of competing interest

The authors declare that they have no known competing financial interests or personal relationships that could have appeared to influence the work reported in this paper.

## Data Availability

No data was used for the research described in the article.
